# Supplementary material for: Umbilical Endometriosis: A Systematic Literature Review and Pathogenic Theory Proposal
Source: J Clin Med. 2022 Feb 14;11(4):995. doi: 10.3390/jcm11040995 (PMC8879338; doi:10.3390/jcm11040995)
Supplement: Supplementary file 1 [file jcm-11-00995-s001.zip › Table S2.pdf]

Author, year	MINORS 1 aim	MINORS 2 consecutive	MINORS 3 prospective	MINORS 4 endpoints	MINORS 5 unbiased	MINORS 6 Follow up	MINORS 7 Loss to FU	MINORS 8 Study size	TOTAL
**Clinical series**									
**Steck WD and Helwing EB, 1965**	2	0	0	2	2	2	0	0	**8**
**McKenna, Wade Evans, 1985**	**2**	**2**	**0**	**2**	**2**	**0**	**0**	**0**	**8**
**Khetan et al, 1999***	**1**	**2**	**0**	**0**	**0**	**2**	**0**	**0**	**6**
**Zhao X *et al*, 2005**	2	2	0	2	2	2	0	0	**10**
**Agarwal A *et al*, 2008**	1	2	0	1	1	2	2	0	**9**
**Leite GK *et al*,, 2009**	2	2	0	2	2	1	1	0	**10**
**Savelli L *et al*, 2012**	2	2	0	2	2	0	0	0	**8**
**Ecker AM *et al*, 2014**	2	2	0	2	2	0	0	0	**8**
**Vellido-Cotelo R *et al*, 2015**	2	2	0	2	2	0	0	0	**8**
**Chrysostomou A *et al*, 2017**	2	2	2	2	2	2	1	0	**13**
**Marras S *et al*, 2019**	2	2	0	2	2	2	1	0	**11**
**Youssef AT *et al* 2020**	2	2	0	2	2	1	0	0	**9**
**Case series**									
**King, 1954***	1	2	0	1	1	0	0	0	5
**Michowitz et al, 1983***	0	2	0	0	0	0	0	0	2
**Rabinovitch *et al*, 1952**	2	0	0	2	2	0	0	0	**6**
**Pathak UN; Hayes JA, 1968**	1	2	0	2	2	2	1	0	**10**
**Lattuneddu *et al*, 2002**	2	2	0	2	2	0	1	0	**9**
**Al-Saad S, 2007**	2	2	0	2	2	2	1	0	**11**
**Dessy *et al*, 2008**	2	2	0	2	2	2	1	0	**11**
**Fedele *et al*, 2010**	2	2	0	2	2	2	1	0	**11**
**Abramowicz *et a*, 2011**	2	2	0	2	2	0	1	0	**9**
**Darouichi *et al*, 2013**	1	2	0	2	2	0	0	0	**7**
**Saito *et al*, 2013**	2	2	0	2	2	2	1	0	**11**
**Chikazawa *et al*, 2014**	1	2	0	2	2	0	0	0	**7**
**Boesgaard-Kjer *et al*, 2017**	2	2	0	2	2	1	0	0	**9**
**Dos Santos Filho *et al*, 2018**	2	2	0	2	2	2	1	0	**11**
**Hirata *et al*, 2020**	2	2	0	2	2	2	1	0	**11**

**Makena *et al*, 2020**	2	2	0	2	2	1	1	0	**10**

* **Excluded for low MINORS score**
